# Qualitative phytochemical profiling, and in vitro antimicrobial and antioxidant activity of *Psidium guajava* (Guava)

**DOI:** 10.1371/journal.pone.0321190

**Published:** 2025-04-07

**Authors:** Mauricia Möwes, Graça K. Kandanda, Loide N. Nangolo, Festus S. Shafodino, Lamech M. Mwapagha

**Affiliations:** Department of Biology, Chemistry and Physics, Faculty of Health, Natural Resources and Applied Sciences, Namibia University of Science and Technology, Private Bag, Windhoek, Namibia; University of Jeddah, Saudi Arabia

## Abstract

*Psidium guajava* is a well-known tropic tree, widely cultivated for its fruits, and traditionally, it has long been used for medicinal purposes. For instance, its fruit peels are also being used to alleviate stomach cramps in Namibia and its leaves-derived aqueous extract are used to treat Candidiasis (yeast infection) caused by *Candida albicans* in some parts of the world. Therefore, this study identified the phytochemical compounds in *Psidium guajava* leaf and fruit peels extracts, determined its antioxidant and antimicrobial activities. *Psidium guajava* leaves and fruit peels extracts were obtained using five solvents (Acetone, methanol, aqueous acetone, aqueous methanol and water) via maceration and boiling extraction methods. The extracts were then subjected to phytochemical screening, Gas chromatography-Mass spectrometry, Fourier Transform Infrared Spectroscopy, antioxidant, and antimicrobial analyses (against pathogenic bacteria: *Escherichia coli, Salmonella spp., Staphylococcus aureus* and fungus; *Candida albicans*). The qualitative phytochemical screening revealed the presence of alkaloids, flavonoids, phenols, tannins, steroids, saponins and terpenoids, and some of their associated functional groups were revealed by Fourier transform infrared spectroscopy analysis. Gas chromatography-Mass spectrometry analysis identified various compounds with antimicrobial and antioxidant properties. The different crude extracts exhibited varying inhibitory effects against the selected pathogens, with the leave extracts exhibiting the highest antimicrobial activity whereas, the peel extract exhibited the highest antioxidant activity. This study thus highlights *Psidium guajava’s* intriguing therapeutic contribution towards the survival of humankind and it can be strategized for future use to treat pathogenic bacterial diseases.

## Introduction

*P. guajava* (commonly known as Guava), a typical tropical tree and food plant, is a member of the Magnoliophyta phylum, Magnoliopsida class, and Myrtaceae family [1], and it is widely distributed across different countries. *P. guajava* contains several bioactive compounds, such as flavonoids, tannins, phenols, alkaloids, triterpenes, saponins, carotenoids, lectins, vitamins, carbohydrate, fibre fatty acids, and glycosides [2,3], which have been shown to improve and stabilize several physiological and metabolic functions in the human body. As a result, *P. guajava* has unique pharmacological and medicinal properties that support a variety of applications as an important plant component in medicinal research and a low-cost ingredient in meals [4].

Depending on the nature of illnesses, different preparations of *P. guajava* are administered orally or topically by various native people [5]. The leaves’ aqueous-based extracts of the *P. guajava* plant is used as traditional medicine to treat Candidiasis (yeast infections) caused by the fungus *Candida albicans*, whereas its fruit peels are used to treat stomach cramps and diarrhoea [6].

It has been reported that over 80% of the world’s population uses medicinal plants and/or their bioactive compounds to prevent, manage or treat a variety of illnesses [5]. This has drawn the attention of many scientific researchers due to their use in the discovery of natural constituents, drug discovery for therapeutics, as well as in the treatment of serious illnesses including cancer, diabetes, and hypertension in an ethnomedical setting. *Psidium guajava* is of no exception, as it is widely used traditionally for treating various diseases, such as, but not limited to diarrhoea, rheumatism, diabetes, digestive problems, laryngitis, ulcers, malaria, cough, bacterial infections, wound healing and pain relief [2,7].

Additionally, *P. guajava* leaves contain a high concentration of essential antioxidants, such as quercetin [4], which is the most active antioxidant in *P. guajava* leaves and is responsible for its spasmolytic effect. Antioxidants are essential for human health and well-being because they can help prevent or slow down cell damage caused by free radicals. Additionally, various other studies [2,3,8,9] reported the abundant presence of essential oils (β-bisabolene, caryophyllene oxide, β-copanene, farnesene, longicyclene, humlene, selinene, cardinene, curcumene, β-caryophyllene, pinene, caryophyllene oxide, 1,8-cineeole and limonene) in the leaves of *P. guajava*.

With increasing bacterial infections and the burden of antimicrobial resistance (AMR), modern medicine is confronted with the threat that antibiotics may lose their efficacy on bacteria and the associated ability to treat bacterial infections [10, 11, 12]. A study done by Sartorius et al. [13] reported that by 2050 approximately 50 million deaths could occur annually due to AMR associated and attributable diseases. Furthermore, the same study showed that there is a disproportionate burden of AMR in both low and middle-income countries (LMICs), especially in sub-Saharan countries which lack effective surveillance systems, laboratory diagnostics and access to crucial or more effective antibiotics. Thus, increasing the AMR burden, morbidity, mortality and health care costs [13]. Cheaper, and more readily available antimicrobials with novel mechanisms of action are needed, especially from natural products to overcome the socio-economic and health impact caused by AMR. Traditional medicine could be advantageous, due to simple availability without a prescription, low cost, natural origin, and the potential to reduce the need for synthetic medications that may have severe adverse effects [14].

It is worth noting that altitude/elevation play major roles in altering the biosynthesis and composition/content of various secondary metabolites and compounds in plants, therefore, the content of these compounds could differ depending on the geographical location [15]. Most parts of the world (including Namibia) boil and utilize the leaves as well as the peels of *P. guava* as traditional curative remedies for various bacterial and yeast infections [13]

Although many studies (conducted in other countries) have documented the medicinal properties of *P. guajava*, continuous scientific research is needed to completely understand its full medicinal potential. Furthermore, because the composition of phytochemical compounds differs depending on geographic location, there is a shortage of data regarding the phytochemical profile of *P. guajava* grown under Namibian conditions.This study sought to bridge this gap by laying a foundation that supports current available literature on the medicinal value of *P. guajava*.

## Methods

### Sample collection and preparation

The *P. guajava* leaves and fruits were collected from Windhoek (located at -22° 33’ 89 33.876’‘ N and 17° 4’ 59.628 E), Namibia. The leaf samples were washed with tap water, shade dried for 3 weeks and pulverized using a mortar, pestle, and a coffee blender (BOSCH 91 MKM6003), then kept until further processing and analysis.

### Extraction of essential compounds from leaves and fruit peels of *Psidium guajava*

Approximately 5 grams of powdered leaf and fruit peel samples were weighed and extracted separately using maceration with 50 ml of each solvent (i.e., Pure methanol, aqueous methanol (70%), pure acetone and aqueous acetone (70%)) for 48 hours at 500rpm and room temperature. As for the aqueous extract preparation, 50 grams of leaves were rinsed with tap water, followed by distilled water, and then boiled in 750 ml distilled water for 4 hours which is a similar approach to the traditional way of doing it. All the obtained extracts were then filtered using Macherey-Nagel filter paper (110mm 99 – REF 431 011) after extraction and the filtrates were concentrated with the rotary evaporator (with water bath temperatures of 55°C for methanol, 46°C for acetone and 60°C for aqueous extracts). The obtained concentrated extracts were stored in reagent bottles in the fridge at 4 °C until further analysis.

### Phytochemical screening of compounds

Standard phytochemical screening methods were used to test the extracts for the presence of active compounds such as alkaloids, tannins, saponins, flavonoids, phenolic compounds, terpenoids and cardiac glycosides, that can exhibit antioxidant and antimicrobial properties [16].

#### Test for alkaloids.

A volume of 1.5 ml of 1% hydrochloric acid (HCl) was added to 2.0 ml of each extract in a test tube. Six drops of Wagner’s reagent were added after heating the test tube content over the water bath. The presence of alkaloids was shown by the formation of an orange precipitate.

#### Test for flavonoids.

A few drops of ferric chloride hexahydrate (FeCl_3_ ∙ 6H_2_O) solution were added to 2.0 ml of each extract. The formation of an intense green colour indicated the presence of flavonoids.

#### Test for phenols.

A few drops of 5% ferric chloride hexahydrate (FeCl_3_ ∙ 6H_2_O) solution were added to 2.0 ml of each extract. The presence of phenols was indicated by a deep blue-black colour.

#### Test for tannins.

A volume of 1.0 ml of each extract was mixed with 2.0 ml of distilled water. To this mixture, 2.0 ml of 5% ferric chloride hexahydrate (FeCl_3_ ∙ 6H_2_O) solution was added, and the resulting brownish-green or dark-green solution confirmed the presence of tannins.

#### Test for cardiac glycosides.

A volume of 3.0 ml of glacial acetic acid (CH_3_COOH) was added to 2.0 ml of each extract in the test tube followed by addition of 1 drop of 5% ferric chloride hexahydrate (FeCl_3_ ∙ 6H_2_O), and 0.5 ml of concentrated sulphuric acid (H_2_SO_4_) added carefully by the side of the test tube. The blue colour formed in CH_3_COOH indicates the presence of cardiac glycosides.

#### Test for steroids.

A volume of 5.0 ml of chloroform (CHCl_3_) and 2.0 ml of acetic anhydride ((CH ₃CO)₂O) were added to 2.0 ml of each extract followed by concentrated H_2_SO_4_. The reddish-brown coloration at the interface indicated the presence of steroids.

#### Test for saponins.

Each extract was diluted with distilled water and shaken in a test tube for 15 minutes. The presence of saponins was indicated by the formation of a layer of foam.

#### Test for terpenoids.

A volume of 2.0 ml of chloroform (CHCl_3_) was mixed with 1.0 ml of extract and 3.0 ml of concentrated Sulfuric acid (H_2_SO_4_) was carefully added to form a layer. The presence of terpenoids was indicated by a reddish-brown coloration at the interface.

### Fourier Transform Infrared Spectroscopy (FT-IR) analysis

The leaves and fruit peel extracts were dried (at 25- 27 ° C), overnight (18-24 hrs) and analysed using the Spectrum Two FT-IR (Perkin Elmer) for the identification of the different functional groups linked to the phytochemicals in the samples that account for antioxidant and antimicrobial properties.

### Compound identification by Gas Chromatography-Mass Spectrometry (GC-MS) analysis

Before subjecting the extracts to GC-MS analysis, they were cleaned up by adding 25 mg of magnesium sulfate and 4.17 mg of silica to 1ml of each extract, followed by vortexing and centrifuging for 10 minutes and the supernatant was then used for analysis. To identify compounds, present in the extracts that may be of antioxidant, antimicrobial and any other medicinal value, a Perkin Elmer CLARUS 680 gas chromatography coupled with a CLARUS SQ 8 mass spectrometer was utilized with the following conditions: column Elite-5MS 30m x 0.25mm x 0.25 µm capillary column, operating at an electron impact mode at 70eV and using helium (99.99%) as the carrier gas at a flow of 11mL/min. An injection volume of 0.2µl was used in a split ratio of 10:1. The oven temperature programmed for 40°C raised to 300°C at a rate of 3°C/min. The compounds were then identified using the National Institute of Standards and Technology (NIST) library [17].

### Antimicrobial property analysis

The following media were prepared, plated, and incubated overnight at 35°C ±  2: Nutrient Agar (for *Escherichia coli* and *Staphylococcus aureus*), Potato Dextrose Agar (for *C. albicans*) and MacConkey Agar (for *Salmonella spp*.). A few colonies (i.e., 2 to 3 colonies) were gently scraped from the freshly prepared cultures and placed into 2ml sterile water. The density of the suspensions was adjusted to 0.5 McFarland (for *E. coli, S. aureus* and *Salmonella spp.)* and 1.0 McFarland (for *C. albicans*) using a Vitek densicheck. A set of new plates was then inoculated with 300 µl of the freshly prepared suspensions containing the test microorganisms (*C. albicans, E. coli, S. aureus* and *Salmonella spp*.). Consequently, sterile filter paper discs (of about 6 mm in diameter) were dipped in the various leaves and peels extracts (acetone, methanol, 70% acetone and methanol, and water) of *P. guajava* and placed on the inoculated agar surface. Distilled water was used as a negative control and six antibiotics (Vancomycin, Tetracycline, Erythromycin, Cefuroxime, Amoxicillin-Clavulanic acid and Ampicillin) were used as positive controls. The Petri dishes were incubated at 35°C ±  2 for 48 hours. The analyses were done in triplicate, and the diameters of inhibition zones were measured using a ruler and compared with American Society for Microbiology standards [18].

### Antioxidant analysis

Total free radical scavenging capacity of the extracts was estimated using the stable 2,2-diphenyl-1-picrylhydrazyl (DPPH) radical, which has an absorption maximum at 515 nm. A solution of the free radical was prepared by dissolving 24 mg DPPH in 100 ml methanol and kept in the dark for at least 30 minutes to allow the free radical formation. Vitamin C stock solution (positive control) was prepared by dissolving 2 mg of ascorbic acid powder in 20 ml of ethanol (99%). A solution series of the extract and positive control were prepared (volume of the extracts and/or positive controls used varied from 1 ml to 5 ml and topped up to 10 ml methanol (99%). Approximately 1 ml of each solution was mixed with 3 ml methanolic DPPH solution and subsequently diluted to the 10 ml mark with 99% methanol. The mixtures were shaken vigorously and kept at room temperature for 30 min in the dark. Absorbance of the reaction mixtures were measured at a wavelength of 515 nm using the UV-Vis spectrophotometer (PerkinElmer). All the determinations were performed in triplicate [19].

The following equation (1) was used to calculate the percentage radical scavenging activity [20]


$$Radicalscavengingactivity\left(\%\right)=\left[\left(Ac-As\right)÷Ac\right]\times 100$$ (1)

where: Ac — Control absorbance; As—Testing specimen absorbance.

The IC50 values were calculated using linear regression equations in which the concentration of the samples was on the x-axis and percent inhibition on the y-axis. From the equation y =  a +  bx (where a is the y-intercept, b is the gradient and x is the concentration), IC50 values were calculated using the following equation (2) [21].


$$IC50=\frac{50-a}{b}$$ (2)

## Results

### Phytochemical screening

The various *P. guajava* leaf and peel extracts were screened for eight phytochemicals that could contribute to the plant’s medicinal value (specifically antioxidant and antimicrobial activity). Overall, seven out of the eight tested phytochemicals were detected with respect to the extracts, namely alkaloids, flavonoids, phenols, tannins, steroids, saponins and terpenoids (Table 1). The anticipated blue colour was not observed for the positive detection of cardiac glycosides.

**Table 1:** Phytochemical screening results of P. guajava leaves and peels extracted with methanol, acetone and aqueous extracts.

Compound ↓	70% methanol		Pure methanol		70% acetone		Pure acetone		Aqueous	Observations
Part of *P. guajava* use →	Leaves	Peels	Leaves	Peels	Leaves	Peels	Leaves	Peels	Leaves	
Alkaloids	+	+	–	+	+	+	–	+	+	Orange precipitate
Flavonoids	–	+	–	–	+	+	+	+	+	Intense green
Phenols	+	–	+	–	+	+	+	+	+	Deep blue-black
Tannins	–	+	+	+	+	+	+	+	+	Brownish green/dark green
Cardiac glycosides	–	–	–	–	–	–	–	–	–	Blue colour
Steroids	+	+	–	+	+	+	–	+	+	Reddish-brown
Saponins	+	+	+	+	+	+	+	+	+	Foam layer
Terpenoids	+	+	+	+	+	+	–	+	+	Reddish-brown

Note: (-) implies the absence of a phytochemical, while (+) implies its presence.

### Fourier transform infrared spectroscopy (FT-IR) analysis

**Fig 1:**
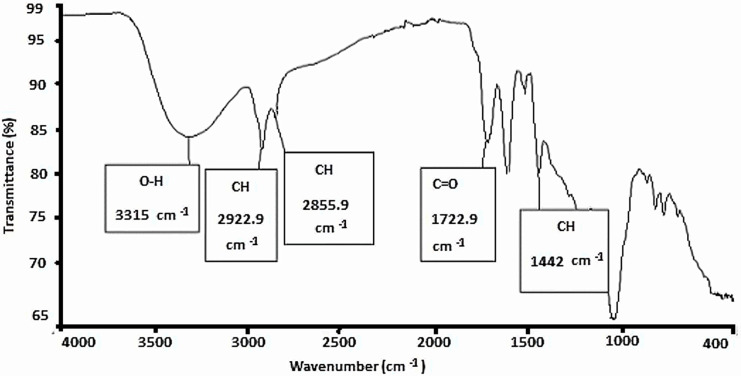
FT-IR spectrum of P. guajava fruit peels’ acetone extract scanned at 4000-400 cm-1, with x-axis: wavenumber (cm-1) and y-axis: transmittance (%T). The spectrum illustrates various existing peaks at specific wavenumbers, corresponding to key functional groups that are present in this extract.

The *P. guajava* samples were analysed to identify specific functional groups correlating to the phytochemicals detected, which are responsible for the antimicrobial and antioxidant activity. The hydroxyl (OH) functional group was identified at the highest frequency range of 2500-3300 cm^-1^and it was followed by C-H stretching at 2850-2950 cm^-1^ which is an alkane functional group. Moreover, carbonyl group (with C = O stretching bond) was identified at a frequency range of 1700-1735 cm^-1^. Lastly, at the lowest frequency range, an alkene functional group of = C-H bending bond was identified at 1370-1450 cm^-1^ (Table 2). Majority of the peaks observed in the various spectra were relatively similar throughout all the spectra of all extracts and they fall within the same frequency range (Fig S1 – S10). The reference spectrum with peaks observed for fruit peels extracted with acetone is illustrated in Fig 1.

**Table 2:** Identified functional groups and correlating phytochemicals of P. guajava, based on FT-IR spectra scanned at 4000-400 cm -1.

Peak nr.	Frequency range (cm-1)	Functional group	Present in	Correlating phytochemicals	Biological activity	References
1	2500-3300	Hydroxyl (O-H stretching)	All extracts and powder samples	Phenols	Anti-inflammatory effects and antimicrobial	[22, 23]
2	2850-2950	Alkanes (C-H stretching)	All extracts and powder samples	Steroids; Terpenoids	Anti-cancer, anti-inflammatory	[24, 25]
3	1700-1735	Carbonyl (C = O stretching)	P100A; P70A; L100M; P70M; L70M; P100M; powder samples	Polyphenols	Anti-inflammatory effects and antimicrobial	[26, 27]
4	1370-1450	Alkenes (=C-H bending)	L100A; L70A; P100A; L100M; L70M; P100M; P70M; WE; PP	Terpenoids	Anti-cancer, anti-inflammatory	[24, 25]

*P100A: peels extracted with pure acetone; P70A: peels extracted with 70% acetone; L100M: leaves extracted with pure methanol; L70M: leaves extracted with 70% methanol; L70A: leaves extracted with 70% acetone; P100M: peels extracted with pure methanol; P70M: peels extracted with 70% methanol; WE: aqueous extract; PP: peel powder.

### Gas chromatography-mass spectrometry (GC-MS) analysis

**Fig 2:**
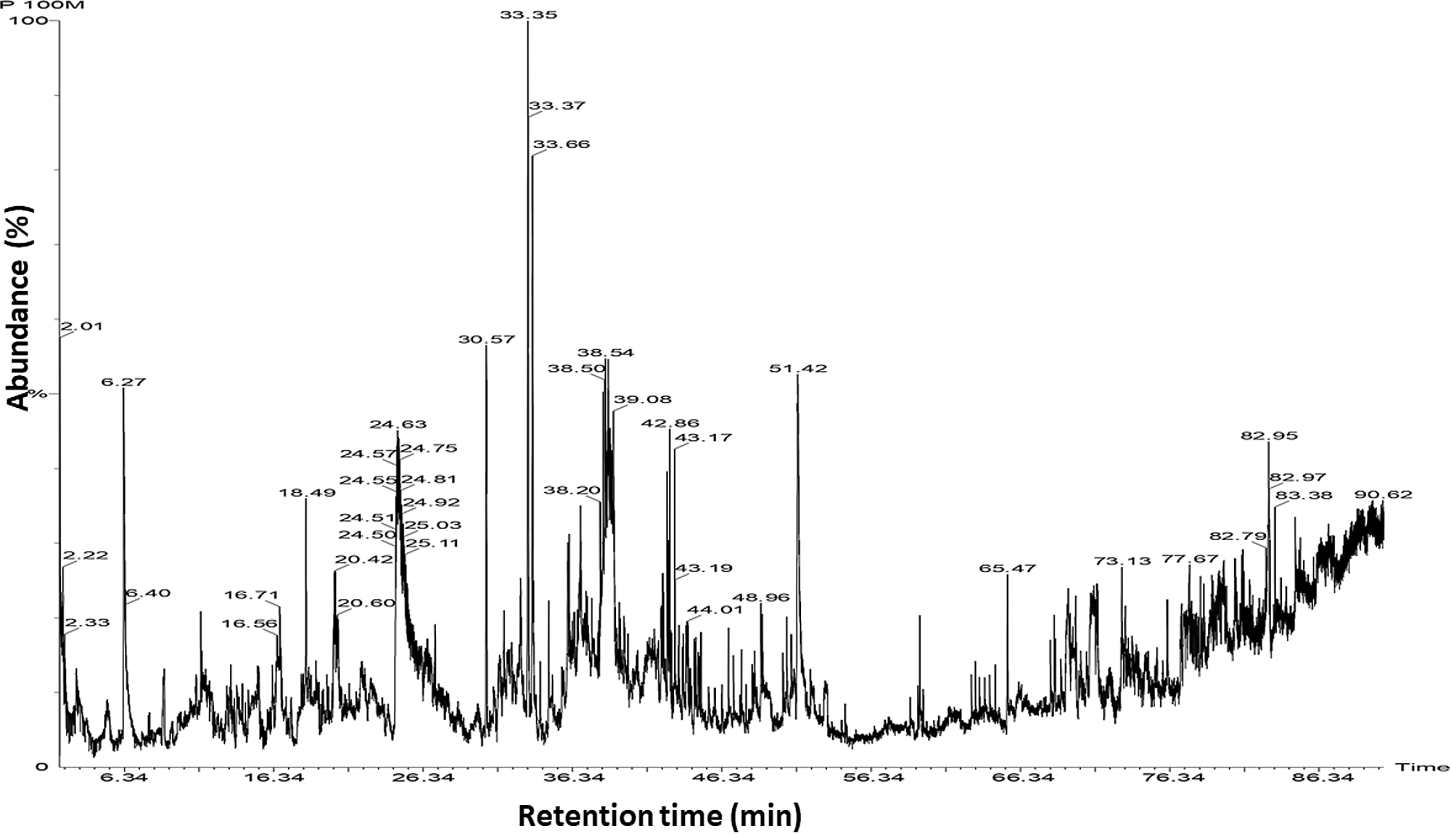
GC-MS chromatogram of P.guajava peels’ methanol extract with x-axis: retention time and y-axis: abundance (%). The chromatogram shows major peaks at various retention times that correspond to different compounds, i.e.,cyclotrisiloxane hexamethyl (2.26 min), Caryophyllene (30.57 min), Bis(2-ethlhexyl) phthalate (65.47 min), Furfural (6.27 min), 5-Hydroxymethylfurfural (24.44 min), 2-Butanone (2.09 min), 2-Pentanone, 4 Hydroxy-4-methyl- (6.74 min), Naphthalene- (33.35 min), Eucalyptol (13.83 min), Bicyclo [5.3.0]Decane- (33.36 min).

GC-MS analysis of the *P. guajava* leaf and peel extracts revealed the presence of ten compounds, out of which nine are known to have antimicrobial and antioxidant properties (Table 3). Peels extracted with pure methanol showed several vital peaks at relative retention times and they were correlated to phytoconstituents known to account for the antimicrobial and antioxidant properties of *P. guajava* (Fig 2). Relatively similar phytochemical compounds were observed throughout all extracts (Fig S11 – S14).

**Table 3:** Compounds identified in Psidium guajava leaf and fruit peel extracts based on GC-MS analysis and their relative biological activities.

Retention time (min)	Rev.	Compound name	Molecular formula	Molecular weight	Present in	Biological activity(s)	Reference(s)
2.26	918	Cyclotrisiloxane, hexamethyl-	C_6_H_18_O_3_Si_3_	222	P70M; P70A	Antioxidant	[28]
30.57	970	Caryophyllene	C_15_H_24_	204	P100M; L100M; L100A	Antimicrobial and antioxidant	[29]
65.47	954	Bis(2-ethylhexyl) phthalate	C_24_H_38_O_4_	390	P100M	Apoptosis (cell death) inhibitor	[30]
6.27	960	Furfural	C_5_H_4_O_2_	96	P100M	Antimicrobial, anti-inflammatory	[31]
24.44	903	5-Hydroxymethylfurfural-	C_6_H_6_O_3_	126	P70A	Antioxidant	[32]
2.09	950	2-Butanone	C_4_H_8_O	72	P100A	Antibacterial	[33]
6.74	940	2-Pentanone,4 Hydroxy-4-methyl-	C_6_H_12_O_2_	116	P100A	Antimicrobial	[34]
33.35	982	Naphthalene-	C_15_H_24_	204	P100A	Antimicrobial and antioxidant	[35]
13.83	960	Eucalyptol	C_10_H_18_O	154	L100A	Antimicrobial, and antioxidant	[36]
33.36	986	Bicyclo[5.3.0]Decane-	C_15_H_24_	204	L100A	Antimicrobial	[37]

*P100A: peels extracted with pure acetone; P70A: peels extracted with 70% acetone; L100M: leaves extracted with pure methanol; L70M: leaves extracted with 70% methanol; L70A: leaves extracted with 70% acetone; L100A: leaves extracted with pure acetone; P100M: peels extracted with pure methanol; P70M: peels extracted with 70% methanol.

### Antimicrobial property analysis

**Fig 3:**
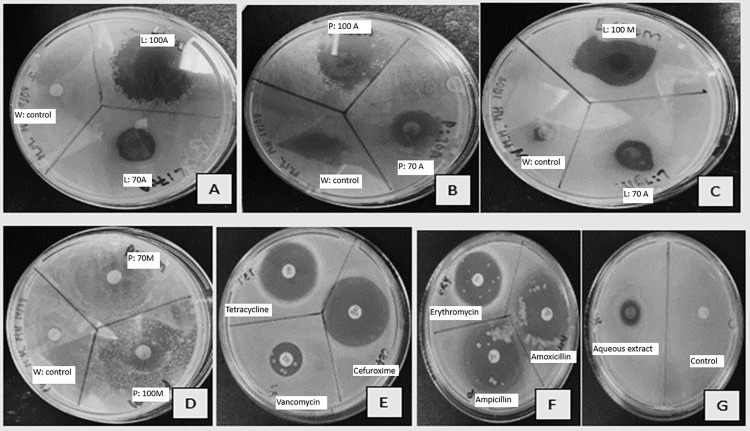
Inhibitory effects of different extracts of Psidium guajava against S.aureus: A: zones of inhibition of leaves extracted with 70% acetone and pure acetone and water as negative control; B: zones of inhibition of peels extracted with 70% acetone and pure acetone and water as negative control; C: zones of inhibition of leaves extracted with 70% methanol and pure methanol and water as control; D: zones of inhibition of peels extracted with 70% methanol and pure methanol and water as control; E: zones of inhibition of Tetracycline, Cefuroxime and Vancomycin; F: zones of inhibition of Erythromycin, Ampicillin and Amoxicillin; G: zones of inhibition of leaves extracted with aqueous extract and distilled water as negative.

The antimicrobial susceptibility test (AST) was done to evaluate the antibacterial and antifungal properties of *P. guajava* (S2 Table*).* The results showing the inhibitory effects of various extracts of *P. guajava* against *S. aureus* are shown in Fig 3. Additionally, the varying zones of inhibition values against the remaining tested microbes are illustrated in Table 4. The 70% methanol leaves extracts exhibited the highest inhibitory effect (9.67 mm ±  1.15) against *E. coli* whereas pure acetone leaves extracts exhibited the highest inhibitory effect (22.33 mm ±  3.21) against *C. albicans* compared to the other extracts. Moreover, *S. aureus* was inhibited (21.00 mm ±  8.19) by leaves extracted with pure acetone and *Salmonella spp*. (14.33 mm ±  0.58) by leaves extracted with 70% acetone. The aqueous extract, prepared as per traditional methods, showed a significant inhibitory effect (9.00 mm ±  7.81) against *C. albicans*. Interestingly, *Salmonella spp*. was the only strain that exhibited resistance against the methanol-based peel extracts.

**Table 4:** Resulting zones of inhibition of Psidium guajava extracts, positive and negative treatments against selected pathogenic microbes.

	Zone of inhibition (mm)			
	*E.coli*	*C.albicans*	*S.aureus*	*Salmonella spp.*
Leaves: 70% acetone	4.00 ± 6.93	15.67 ± 2.31	16.67 ± 2.52	14.33 ± 0.58
Leaves: pure acetone	4.00 ± 6.93	22.33 ± 3.21	21.00 ± 8.19	13.33 ± 2.31
Peels: 70% acetone	8.67 ± 0.58	15.33 ± 6.51	15.00 ± 1.00	11.33 ± 1.15
Peels: pure acetone	5.33 ± 4.62	13.67 ± 3.06	16.33 ± 10.21	11.67 ± 0.58
Leaves: 70% methanol	9.67 ± 1.15	15.33 ± 1.53	13.00 ± 5.00	14.33 ± 3.21
Leaves: pure methanol	8.67 ± 0.58	17.33 ± 0.58	18.00 ± 7.00	14.00 ± 2.65
Peels: 70% methanol	6.67 ± 6.11	10.67 ± 1.53	14.67 ± 3.79	–
Peels: pure methanol	6.67 ± 5.86	12.67 ± 4.62	10.00 ± 1.73	–
Leaves: aqueous extract	7.00 ± 1.00	9.00 ± 7.81	12.33 ± 0.58	6.33 ± 5.51
Negative control (distilled water)	–	–	–	–
Vancomycin	–	–	15.00 ± 0.00	–
Erythromycin	9.00 ± 0.00	9.00 ± 0.00	22.00 ± 0.00	11.00 ± 0.00
Ampicillin	19.00 ± 0.00	–	33.00 ± 0.00	14.00 ± 0.00
Tetracycline	15.00 ± 0.00	–	27.00 ± 0.00	23.00 ± 0.00
Amoxicillin	21.00 ± 0.00	–	26.00 ± 0.00	21.00 ± 0.00
Cefuroxime	22.00 ± 0.00	–	32.00 ± 0.00	18.00 ± 0.00

Note: (-) implies no zone of inhibition observed.

The values are presented in triplicates as mean ±  standard deviation.

### Antioxidant property analysis

The DPPH assay was employed to evaluate the antioxidant activity of *P. guajava* leaf and peel samples (S1 Table), which were subsequently compared against an established category that defines the strength of antioxidant activity (level of antioxidant strength is divided into four levels, which are very strong (IC50 <  50), strong (IC50: 50–100), moderate (IC50: 101–150), and weak (IC50: 250–500) [38]. The IC50 values were recorded in Table 5. The strongest IC50 value obtained from the extracts analysed was 0.78 µl/ml for peels extracted with pure methanol and lowest value being 88.30 µl/ml for both leaves and peels extracted with pure acetone.

**Table 5:** Antioxidant activity of various Psidium guajava extracts and the corresponding standardized categories for antioxidant strength.

Sample	IC_50_ (µl/ml)	Category of antioxidant activity [38].
Leaves: water	3.19	Very strong
Leaves: 70% acetone	4.49	Very strong
Peels: 70% acetone	36.16	Very strong
Peels: pure acetone	0.78	Very strong
Leaves: 70% methanol	22.66	Very strong
Leaves: pure methanol	11.18	Very strong
Leaves: pure acetone	0.78	Very strong
Peels: 70% methanol	23.97	Very strong
Peels: pure methanol	88.30	Strong

## Discussion

### Phytochemical screening

The results revealed the presence of various essential phytochemical compounds in the different *P. guajava* extracts. It is worth highlighting that only the leaves were extracted with the aqueous solvent via boiling, to align with its traditional usage and make the study more relevant in the context of traditional medicine. *P. guajava* extracts were found to contain a broad range of phytochemicals; the 70% methanol extract had the most extensive profile, comprising alkaloids, flavonoids, phenols, tannins, steroids, saponins, and terpenoids. On the other hand, alkaloids and flavonoids were absent from pure methanol extracts, while seven of the eight phytochemicals were detected in both acetone and aqueous extracts. Notably, none of the extracts contained cardiac glycosides. The presence of flavonoids, phenols, and tannins in the 70% methanol extract is particularly significant, as these compounds are well-documented for their potent antioxidant and antimicrobial properties [39].

Another study also noted that cardiac glycosides were absent in *P. guajava* samples and established that environmental and geographical factors could be reason behind this [40]. This study also revealed the presence of similar medically important phytochemicals in guava leaves extracts. In a separate study, cardiac glycosides were detected in acetone extracts contrary to the results of the current study and it was opined that the time of extraction and solid-liquid ratios can account for these variations. Moreover, a low concentration of the tested cardiac glycosides in the tested extracts can often not be detected especially when it is below the detection limit of the analysis method and/or technique employed [41].

It was previously established that flavonoids can be extracted using pure methanol unlike in the current study although detected in other extracts that were assayed (i.e., acetone). The effectiveness of the extraction of flavonoids by methanol, ethanol, and acetone has been evaluated and it was confirmed that acetone is a better extractant than methanol [42], as it is evident with the results of the current study (Table 1). Additionally, it has been reported by Shikwambi et al. [43] that different solvents can lead to varied phytochemical concentrations. This study further reported that addition of water to alcoholic solvent can improve the polarity of the resulting solvent and enhance its extraction efficiency for polar phytochemicals like flavonoids from the herbal matrix. For example, aqueous methanol can be better at isolating flavonoids than pure methanol and/or water in general.

Phytochemical compounds occur naturally in different parts of plants as secondary metabolites They can play diverse roles ranging from serving as feeding defence against predators and providing structural support to plants. In addition to antioxidant and antimicrobial properties, these phytochemicals have been well documented to also collectively elicit various other pharmacological and biochemical properties such as anticancer, analgesic, antifungal, antidiarrheal, anti-hemorrhoidal, anti-inflammatory, cardiac depressant, and hyper-cholesterolemic [40]. Thus, contributing to the medicinal value of *Psidium guajava* leaves and fruit peels.

### FT-IR analysis

The FT-IR spectrum of *P. guajava* extracted using absolute acetone was scanned at the mid infrared region of 400 to 4000 cm^-1^to identify the functional groups present and associated with the phytochemicals in *P. guajava,* as illustrated by Fig 1. The complex character of the *P. guajava* extracts is reflected by several peaks illustrated within the spectrum. Four significant functional groups were identified in *P. guajava* extracts through FTIR analysis such as the hydroxyl groups (OH) at 2500-3300 cm ⁻ ¹, alkanes (C-H stretching) at 2850-2950 cm ⁻ ¹, carbonyl groups (C = O stretching) at 1700-1735 cm ⁻ ¹, and alkenes (=C-H bending) at 1370-1450 cm ⁻ ¹. A previous study established similar findings with phenols, as the hydroxyl functional group could be identified at the highest peak and frequency range of 2500-3000 cm-1 [22]. Moreover, the peak was consistent and relatively similar in all the spectra of the extracts, showing possible antioxidant properties. This can be correlated to the high antioxidant activity observed in the 70% methanol extract where phenols were prominently identified.

Alkane and alkene functional groups were identified at the second highest peak and ranging in frequencies of 2850-2950 cm^-1^ and 1370-1450, respectively, further supporting the presence of terpenoids and other aliphatic compounds, which are associated with antimicrobial properties. Polyphenols, having known anti-inflammatory and antimicrobial properties, could be linked to the frequency range of 1700 to 1735 cm^-1^ [24]. Finally, the detection of carbonyl functional groups (C = O) aligns with the presence of tannins and flavonoids, compounds that contribute to both antioxidant and antimicrobial activities.

An alternative study confirmed that the presence of water-soluble compounds, such as terpenoids is illustrated by the intensity decay and shift of aliphatic C-H stretching and bending mode peaks to low frequency regions at approximately 3000 and 1400 cm^-1^. This suggests that *P. guajava* extracts have the capacity to perform dual functions of stabilization and reduction [43]. The hydroxyl groups’ ability to donate hydrogen atoms makes them crucial in neutralizing free radicals, thereby enhancing the antioxidant activity of the extracts. Similarly, the carbonyl group’s ability to interact with microbial cell walls could explain the antimicrobial efficacy observed. The presence of alkene functional groups, particularly in terpenoids, suggests a role in disrupting microbial cell membranes, further contributing to the antimicrobial properties of *Psidium guajava* extracts.

The identification of these functional groups not only confirms the presence of key bioactive compounds but also highlights the chemical foundations underlying the antioxidant and antimicrobial activities of *Psidium guajava* extracts. These findings support the potential use of *P. guajava* in developing natural therapeutic agents, emphasizing the importance of these functional groups in the plant’s bioactivity.

### GC-MS analysis

The GC-MS analysis revealed several bioactive compounds: Cyclotrisiloxane, hexamethyl- was identified in both aqueous methanol and acetone extracts; Caryophyllene was present in both acetone and methanol extracts; Bis(2-ethylhexyl) phthalate and Furfural were found in the methanol extract; while the acetone extract contained 5-Hydroxymethylfurfural, 2-Butanone, 2-Pentanone, 4-Hydroxy-4-methyl-, Naphthalene, Eucalyptol, and Bicyclo[5.3.0]Decane.Cyclic, unsaturated cyclotrisiloxane, hexamethyl has been reported to exhibit antibacterial and antioxidant activities [44]. Moreover, caryophyllene was identified in several extracts analysed and has shown antioxidant properties in a previous study as well [45]. The presence of Caryophyllene, a known antimicrobial and anti-inflammatory compound, across multiple extracts suggests it plays a significant role in the antimicrobial activity observed in *P. guajava*. Similarly, Eucalyptol, identified in the acetone extract, is recognized for its antioxidant and antimicrobial properties, contributing to the dual bioactivity of the extract. Other studies (Ismail et al. and Jassal et al. 2021) [45, 46] also concurred with these results and additionally reported its antiseptic and anti-inflammatory properties [46]. The identification of 5-Hydroxymethylfurfural, known for its antioxidant potential, aligns with the strong antioxidant activity detected in the acetone extract. The identification of Bis(2-ethylhexyl) phthalate and Furfural in methanol extract, both of which have industrial relevance and moderate bioactivity, may contribute to its distinct profile in antimicrobial and antioxidant assays. In contrast, the acetone extract’s richer variety of bioactive compounds, including Eucalyptol and Naphthalene, likely enhances its broader spectrum of antimicrobial and antioxidant effects.

In contrast, one study reported 30 compounds in *P. guajava* leaves [47]. Similarly, other studies led to the identification of 54 and 64 chemical compounds respectively in oil extracted from *P. guajava* leaves [3,46]. Another study compared the volatile organic compounds (VOCs) profile of two different *P. guajava* species grown in two different countries (Egypt and India) and identified a total of 42 different VOCs. They attributed the observed differences in VOCs profile/composition to geographic origin and growing habitats/conditions of the two species [48].

These GC-MS findings provide a detailed chemical basis for the observed bioactivities in *P. guajava* extracts. The identification of multiple bioactive compounds highlights the plant’s potential as a source of natural antimicrobial and antioxidant agents, supporting its use in developing health-related products. The diverse chemical profile, particularly in the acetone extract, underscores the importance of solvent choice in maximizing the extraction of beneficial compounds.

### Antimicrobial property analysis

The study demonstrated that various extracts of *P. guajava* leaves and fruit peels exhibited significant antimicrobial activity against *E. coli, S. aureus, Salmonella spp., and C. albicans.* Notably, the aqueous extract effectively inhibited *C. albicans*, a pathogen responsible for candidiasis, while *Salmonella spp.* showed resistance to both methanol and aqueous methanol fruit peel extracts.

The antimicrobial efficacy of these extracts can be attributed to the presence of specific phytochemicals and functional groups identified in the study. For instance, the strong inhibition of *C. albicans* by the aqueous extract aligns with the presence of hydroxyl groups (OH) and carbonyl groups (C = O), which are commonly associated with antimicrobial properties [49]. Moreover, just like conventional antimicrobials, these phytochemicals also exhibit specific clinically relevant mechanisms of action against a wide range of pathogenic microbes. For instance, the primary antibacterial mechanisms of alkaloids are suppression of bacterial cell wall development, modification of cell membrane permeability, inhibition of bacterial metabolism, and inhibition of nucleic acid and protein synthesis [49]. Similarly, tannins (identified in the 70% methanol extract) are ligands that can bind to proteins via hydrophobic interactions and hydrogen bonding. As a result, the metabolism of bacteria is inhibited [50]. On the other hand, flavonoids (also identified in the 70% methanol extract) have the following antibacterial mechanisms: inhibition of nucleic acid synthesis, inhibition of cytoplasmic membrane function, inhibition of energy metabolism, inhibition of attachment and biofilm formation, inhibition of the porin on the cell membrane, alteration of membrane permeability, and attenuation of pathogenicity [51]. A similar study also noted that the mechanisms of action of phenols on bacterial cells have been linked to membrane damage, inhibition of virulence factors such as enzymes and toxins, and inhibition of bacterial biofilm formation [52].

The mechanism of the steroids’ antibacterial effect can be explained by the fact that they are similar to steroids that are regularly employed in bacterial cells and replace and/or imitate these chemicals in the cell membrane [53]. Moreover, saponins inhibit bacteria through a variety of techniques. Most notably, they have been found to interact with cell membrane cholesterol, forming a pore and eventually causing the cell membrane to break [54]. Terpenoids primarily exploit their lipophilicity to damage bacterial cell membranes. Terpenoids can penetrate through bacteria’s phospholipid bilayer and diffuse within, exhibiting antibacterial or bactericidal actions. They also prevent the creation of ATP and proteins [55]. Thus, collectively contributing to the observed zones of inhibition against the selected pathogenic microbes.

Additionally, the GC-MS analysis revealed the presence of compounds such as Caryophyllene and Eucalyptol, both known for their antimicrobial properties. Caryophyllene, found in the acetone and methanol extracts, likely plays a role in the observed antibacterial activity against *E. coli* and *S. aureus*. The presence of Eucalyptol in the acetone extract supports its traditional use in treating candidiasis, as this compound is recognized for its efficacy against fungal pathogens like *C. albicans* [13].

It has been established by a study that susceptible bacteria and fungi are defined as those having inhibition zones larger than 9 mm [56]. Therefore, according to the Kirby-Bauer Disk Diffusion Susceptibility Test Protocol of American Society for Microbiology, the observed zone of inhibition demonstrated the effectiveness of *P. guajava’s* extracts against all tested microorganisms (except for methanolic peel extracts used against *Salmonella spp*.). This observed exception could be attributed to the ineffective mechanism of action of the phytochemicals present in the methanolic peel extracts against S*almonella spp*. For example, alkaloids (identified in the methanolic extracts), have a similar mode of action to Vancomycin, they target and penetrate the cell wall of microorganisms*, and Salmonella spp*., having a thick cell wall and asymmetrical outer membrane, combats this penetration, exhibiting an intrinsic resistance [57].

These findings validate the traditional use of *P. guajava* fruit peels for treating stomach cramps and leaves for candidiasis, as the identified phytochemicals and compounds exhibit significant antimicrobial properties [58], as observed by the various zones of inhibitions. However, the resistance of *Salmonella spp.* to both methanol and aqueous methanol peel extracts suggests that further research is needed to optimize extract formulations for broader antimicrobial applications. Overall, this study underscores the potential of *P. guajava* as a natural source of antimicrobial agents, supporting its continued use in traditional medicine and its potential integration into modern therapeutic practices.

### Antioxidant analysis

The antioxidant activity of various *Psidium guajava* extracts was evaluated through IC50 values (measurements were based on the volume of undried extract used, as the concentration of the extract was unknown) with results ranging from 0.78 to 88.30 µl/ml. Notably, the acetone extracts of both leaves and fruit peels exhibited very strong antioxidant activity, with IC50 values of 0.78 µl/ml. Conversely, the methanol extract of fruit peels showed weaker activity, with an IC50 value of 88.30 µl/ml. Many of the extracts demonstrated very strong antioxidant activity, falling well below the 50 µl/ml threshold. The strong antioxidant activity observed in the acetone extracts of *P. guajava* leaves and fruit peels can be attributed to the presence of phenolic compounds, such as flavonoids and tannins, which were identified in these extracts. These compounds are known for their ability to donate hydrogen atoms, thereby neutralizing free radicals. The hydroxyl groups (OH) identified in the FT-IR analysis further support this, as they are associated with the antioxidant potential of phenolic compounds.

Additionally, the GC-MS analysis revealed the presence of bioactive compounds such as Caryophyllene and Eucalyptol, which are known to possess antioxidant properties [28,29]. Caryophyllene, detected in the acetone and methanol extracts, likely contributes to the antioxidant activity of these extracts. The relatively weaker antioxidant activity of the methanol extract of fruit peels (IC50 of 88.30 µl/ml) may be due to the lower concentration of these active compounds, highlighting the importance of solvent selection in maximizing antioxidant potential.

These findings highlight the potent antioxidant activity of *Psidium guajava* extracts, particularly those prepared with acetone, supporting the traditional use of the plant in managing oxidative stress-related conditions. The strong antioxidant activity of the leaf extracts also underscores their potential in treating candidiasis, as oxidative stress is known to play a role in fungal infections. These results suggest that *P. guajava* could be further explored as a natural source of antioxidants for therapeutic applications, particularly in the development of treatments for oxidative stress-related diseases.

## Conclusion

This study offers a comprehensive assessment of the phytochemical profile and in vitro antimicrobial and antioxidant activities of *P. guajava* extracts, which are traditionally used to treat stomach cramps and candidiasis. The research identified a diverse range of compounds and/or phytochemicals (as well as their relative functional groups), including namely alkaloids, flavonoids, phenols, tannins, steroids, saponins and terpenoids, which were identified across various extracts via phytochemical screening, FT-IR and GC-MS analysis. These compounds were associated with the significant antimicrobial and antioxidant activities observed, particularly in the acetone extracts, which exhibited very strong antioxidant activity with IC50 values as low as 0.78 µl/ml.

The antimicrobial analysis demonstrated that *P. guajava* extracts effectively inhibited pathogens such as *E. coli*, *S. aureus*, *Salmonella spp* and *C. albicans*, aligning with the plant’s traditional medicinal uses. However, *Salmonella spp*. showed resistance to certain extracts, indicating variability in antimicrobial efficacy depending on the extract type and pathogen.

While these findings underscore the potential of *P. guajava* as a natural source of antimicrobial and antioxidant agents, the study had certain limitations which are noteworthy, The GC-MS analysis was qualitative because there were no standards available to quantify the number of phytochemicals. These limitations suggest that future research should focus on quantitative analysis to better understand the concentration of bioactive compounds and their specific contributions to the observed activities. Moreover, the study focused only on the leaves and fruit peels of *P. guajava*. Further studies could aim at investigating the phytochemical profile of other parts (e.g., bark and roots) or the entire plant, to provide a more comprehensive understanding of the phytochemical activity.

In line with the results of the current study and several reports from literature, *P. guajava* holds significant promise for developing natural therapeutic agents, particularly for conditions related to oxidative stress and microbial infections. To fully harness its potential, further studies should aim to quantify the identified phytochemicals and explore the standardization of GC-MS analysis, which would enhance the reliability and applicability of the findings in practical and clinical settings. Additionally, future research endeavours should focus on investigating the toxicity profiles, in vivo clinical efficacy, and health risk assessments of the *P. guajava’s* extracts to draw reliable conclusions. These recommended tests can serve as the green light to the final stage of the journey in discovering new and improved antibacterial and antioxidant agents from the extracts.

## Supporting information

S1 Table Absorbance values established using the with the UV-Vis spectrophotometer and subsequent determined total free radical scavenging capacity (%). (DOCX)

S2 Table The triplicate values behind the means and standard deviations of the zones of inhibition (mm) of different P. guajava extracts against various pathogens. (DOCX)

S1 Fig FT-IR spectrum of P. guajava fruit peels’ powder scanned at 4000-400 cm-^1^, with x-axis: wavenumber (cm-^1^) and y-axis: transmittance (%T). The spectrum illustrates various existing peaks at specific wavenumbers, corresponding to key functional groups that are present in this extract.
(DOCX)

S2 Fig FT-IR spectrum of P. guajava leaves’ powder scanned at 4000-400 cm-^1^, with x-axis: wavenumber (cm-^1^) and y-axis: transmittance (%T). The spectrum illustrates various existing peaks at specific wavenumbers, corresponding to key functional groups that are present in this extract.
(DOCX)

S3 Fig FT-IR spectrum of P. guajava fruit peels’ pure methanol extract scanned at 4000-400 cm-^1^, with x-axis: wavenumber (cm-^1^) and y-axis: transmittance (%T). The spectrum illustrates various existing peaks at specific wavenumbers, corresponding to key functional groups that are present in this extract.
(DOCX)

S4 Fig FT-IR spectrum of P. guajava fruit peels’ aqueous methanol extract scanned at 4000-400 cm-^1^, with x-axis: wavenumber (cm-^1^) and y-axis: transmittance (%T). The spectrum illustrates various existing peaks at specific wavenumbers, corresponding to key functional groups that are present in this extract.
(DOCX)

S5 Fig FT-IR spectrum of P. guajava leaves’ aqueous extract scanned at 4000-400 cm-^1^, with x-axis: wavenumber (cm-^1^) and y-axis: transmittance (%T). The spectrum illustrates various existing peaks at specific wavenumbers, corresponding to key functional groups that are present in this extract.
(DOCX)

S6 Fig FT-IR spectrum of P. guajava leaves’ pure acetone extract scanned at 4000-400 cm-^1^, with x-axis: wavenumber (cm-^1^) and y-axis: transmittance (%T). The spectrum illustrates various existing peaks at specific wavenumbers, corresponding to key functional groups that are present in this extract.
(DOCX)

S7 Fig FT-IR spectrum of P. guajava leaves’ aqueous acetone extract scanned at 4000-400 cm-^1^, with x-axis: wavenumber (cm-^1^) and y-axis: transmittance (%T). The spectrum illustrates various existing peaks at specific wavenumbers, corresponding to key functional groups that are present in this extract.
(DOCX)

S8 Fig FT-IR spectrum of P. guajava fruit peels’ pure acetone extract scanned at 4000-400 cm-^1^, with x-axis: wavenumber (cm-^1^) and y-axis: transmittance (%T). The spectrum illustrates various existing peaks at specific wavenumbers, corresponding to key functional groups that are present in this extract.
(DOCX)

S9 Fig FT-IR spectrum of P. guajava fruit peels’ aqueous acetone extract scanned at 4000-400 cm-^1^, with x-axis: wavenumber (cm-^1^) and y-axis: transmittance (%T). The spectrum illustrates various existing peaks at specific wavenumbers, corresponding to key functional groups that are present in this extract.
(DOCX)

S10 Fig FT-IR spectrum of P. guajava leaves’ pure methanol extract scanned at 4000-400 cm-^1^, with x-axis: wavenumber (cm-^1^) and y-axis: transmittance (%T). The spectrum illustrates various existing peaks at specific wavenumbers, corresponding to key functional groups that are present in this extract.
(DOCX)

S11 Fig FT-IR spectrum of P. guajava leaves’ aqueous methanol extract scanned at 4000-400 cm-^1^, with x-axis: wavenumber (cm-^1^) and y-axis: transmittance (%T). The spectrum illustrates various existing peaks at specific wavenumbers, corresponding to key functional groups that are present in this extract.
(DOCX)

S12 Fig GC-MS chromatogram of P. guajava leaves’ pure acetone extract with x-axis: retention time (min) and y-axis: abundance (%). The chromatogram shows major peaks at various retention times that correspond to different compounds.
(DOCX)

S13 Fig GC-MS chromatogram of P. guajava leaves’ pure methanol extract with x-axis: retention time (min) and y-axis: abundance (%). The chromatogram shows major peaks at various retention times that correspond to different compounds.
(DOCX)

S14 Fig GC-MS chromatogram of P. guajava peels’ aqueous acetone extract with x-axis: retention time (min) and y-axis: abundance (%). The chromatogram shows major peaks at various retention times that correspond to different compounds.
(DOCX)
