# Supplementary material for: Qualitative phytochemical profiling, and in vitro antimicrobial and antioxidant activity of Psidium guajava (Guava)
Source: PLoS One. 2025 Apr 7;20(4):e0321190. doi: 10.1371/journal.pone.0321190 (PMC11975133; doi:10.1371/journal.pone.0321190)
Supplement: S1 Table — (DOCX) [file pone.0321190.s001.docx]

**S1 Table: Absorbance values established using the with the UV-Vis spectrophotometer**

| **Sample** | **Absorbance (515 nm)** |
| --- | --- |
| Blank | 1.5621 |
| WE:1 ml | 0.3952 |
| WE: 2 ml | 0.5928 |
| WE:3 ml | 0.6340 |
| WE: 4 ml | 0.9914 |
| WE: 5 ml | 1.0774 |
| L70A: 1 ml | 0.2504 |
| L70A: 2 ml | 0.3599 |
| L70A: 3 ml | 0.5862 |
| L70A: 4 ml | 0.6917 |
| L70A: 5 ml | 0.7742 |
| P70A: 1 ml | 0.0309 |
| P70A: 2 ml | 0.0578 |
| P70A: 3 ml | 0.0806 |
| P70A: 4 ml | 0.0942 |
| P70A: 5 ml | 0.0888 |
| P100A: 1 ml | 0.0228 |
| P100A: 2 ml | 0.0235 |
| P100A: 3 ml | 0.0229 |
| P100A: 4 ml | 0.0237 |
| P100A: 5 ml | 0.0289 |
| L70M: 1 ml | 0.0817 |
| L70M: 2 ml | 0.1206 |
| L70M: 3 ml | 0.1589 |
| L70M: 4 ml | 0.1759 |
| L70M: 5 ml | 0.0581 |
| L100M: 1 ml | 0.1513 |
| L100M: 2 ml | 0.2332 |
| L100M: 3 ml | 0.2948 |
| L100M: 4 ml | 0.3335 |
| L100M: 5 ml | 0.4052 |
| L100A: 1 ml | 0.1040 |
| L100A: 2 ml | 0.2264 |
| L100A: 3 ml | 0.3221 |
| L100A: 4 ml | 0.3435 |
| L100A: 5 ml | 0.3504 |
| P70M: 1 ml | 0.0490 |
| P70M: 2 ml | 0.0710 |
| P70M: 3 ml | 0.1084 |
| P70M: 4 ml | 0.1434 |
| P70M: 5 ml | 0.1380 |
| P100M: 1 ml | 0.0190 |
| P100M: 2 ml | 0.0574 |
| P100M: 3 ml | 0.0513 |
| P100M: 4 ml | 0.0496 |
| P100M: 5 ml | 0.0632 |
| Ascorbic 1 ml | 0.0133 |
| Ascorbic 2 ml | 0.0122 |
| Ascorbic 3 ml | 0.0109 |
| Ascorbic 4 ml | 0.0097 |
| Ascorbic 5 ml | 0.0101 |

*WE= aqueous extract, L70A= leaves extracted with aqueous acetone,P70A = peels extracted with aqueous acetone, P100A = peels extracted with pure acetone, L70M = leaves extracted with aqueous methanol, L100M = leaves extracted with pure methanol, L100A = leaves extracted with pure acetone, P70M = peels extracted with aqueous methanol, P100M = peels extracted with pure methanol
