# Supplementary material for: Qualitative phytochemical profiling, and in vitro antimicrobial and antioxidant activity of Psidium guajava (Guava)
Source: PLoS One. 2025 Apr 7;20(4):e0321190. doi: 10.1371/journal.pone.0321190 (PMC11975133; doi:10.1371/journal.pone.0321190)
Supplement: S2 Table — (DOCX) [file pone.0321190.s002.docx]

**S2 Table. The triplicate values behind the means and standard deviations of the zones of inhibition (mm) of different P. guajava extracts against various pathogens.**

| **Pathogen(s) ↓** | Extract (s) ↓ | Zone of inhibition (mm) | | |
| --- | --- | --- | --- | --- |
| **No of trials →** |  | 1 | 2 | 3 |
| *C. albicans* | Leaves: aqueous acetone | 17 | 17 | 13 |
|  | Leaves: pure acetone | 21 | 26 | 20 |
|  | Peels: aqueous acetone | 22 | 9 | 15 |
|  | Peels: pure acetone | 17 | 13 | 11 |
|  | Leaves: aqueous methanol | 15 | 14 | 17 |
|  | Leaves: pure methanol | 17 | 18 | 17 |
|  | Peels: aqueous methanol | 9 | 11 | 12 |
|  | Peels: pure methanol | 10 | 18 | 10 |
| ***E. coli*** | Leaves: aqueous acetone | 0 | 0 | 12 |
|  | Leaves: pure acetone | 0 | 0 | 12 |
|  | Peels: aqueous acetone | 8 | 9 | 9 |
|  | Peels: pure acetone | 8 | 0 | 8 |
|  | Leaves: aqueous methanol | 9 | 9 | 11 |
|  | Leaves: pure methanol | 8 | 9 | 9 |
|  | Peels: aqueous methanol | 0 | 12 | 8 |
|  | Peels: pure methanol | 11 | 0 | 9 |
| ***S. aureus*** | Leaves: aqueous acetone | 17 | 14 | 19 |
|  | Leaves: pure acetone | 14 | 30 | 19 |
|  | Peels: aqueous acetone | 16 | 14 | 15 |
|  | Peels: pure acetone | 28 | 9 | 12 |
|  | Leaves: aqueous methanol | 18 | 13 | 8 |
|  | Leaves: pure methanol | 15 | 16 | 13 |
|  | Peels: aqueous methanol | 13 | 12 | 19 |
|  | Peels: pure methanol | 9 | 12 | 9 |
| ***Salmonella spp.*** | Leaves: aqueous acetone | 15 | 14 | 14 |
|  | Leaves: pure acetone | 12 | 16 | 12 |
|  | Peels: aqueous acetone | 12 | 10 | 12 |
|  | Peels: pure acetone | 12 | 12 | 11 |
|  | Leaves: aqueous methanol | 18 | 13 | 12 |
|  | Leaves: pure methanol | 15 | 16 | 11 |
|  | Peels: aqueous methanol | 0 | 0 | 0 |
|  | Peels: pure methanol | 0 | 0 | 0 |
