# Supplementary material for: Qualitative phytochemical profiling, and in vitro antimicrobial and antioxidant activity of Psidium guajava (Guava)
Source: PLoS One. 2025 Apr 7;20(4):e0321190. doi: 10.1371/journal.pone.0321190 (PMC11975133; doi:10.1371/journal.pone.0321190)
Supplement: S7 Fig — The spectrum illustrates various existing peaks at specific wavenumbers, corresponding to key functional groups that are present in this extract. (DOCX) [file pone.0321190.s009.docx]

S7 Fig
