# Supplementary figures and images for: Qualitative phytochemical profiling, and in vitro antimicrobial and antioxidant activity of Psidium guajava (Guava)
Source: PLoS One. 2025 Apr 7;20(4):e0321190. doi: 10.1371/journal.pone.0321190 (PMC11975133; doi:10.1371/journal.pone.0321190)

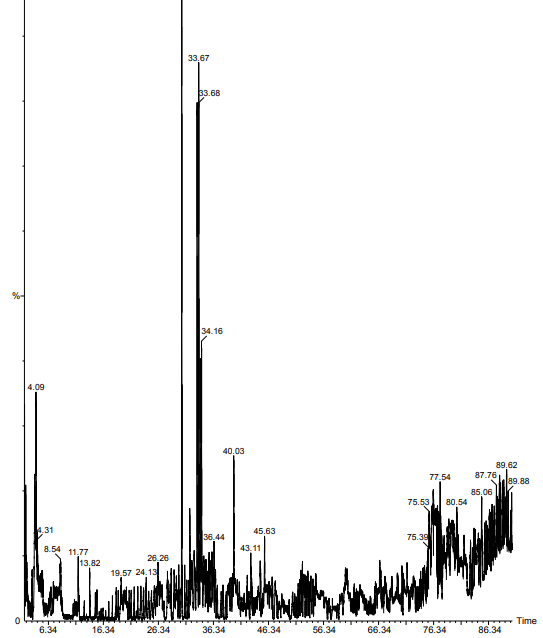


**Abundance (%)**

**Retention time (min)**

S11 Fig

Supplement: S11 Fig — The spectrum illustrates various existing peaks at specific wavenumbers, corresponding to key functional groups that are present in this extract. (DOCX) [file pone.0321190.s013.docx]

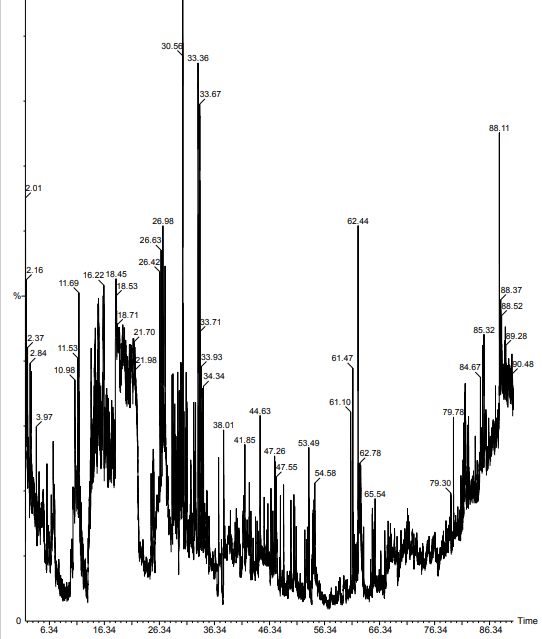


**Abundance (%)**

**Retention time (min)**

S12 Fig

Supplement: S12 Fig — The chromatogram shows major peaks at various retention times that correspond to different compounds. (DOCX) [file pone.0321190.s014.docx]

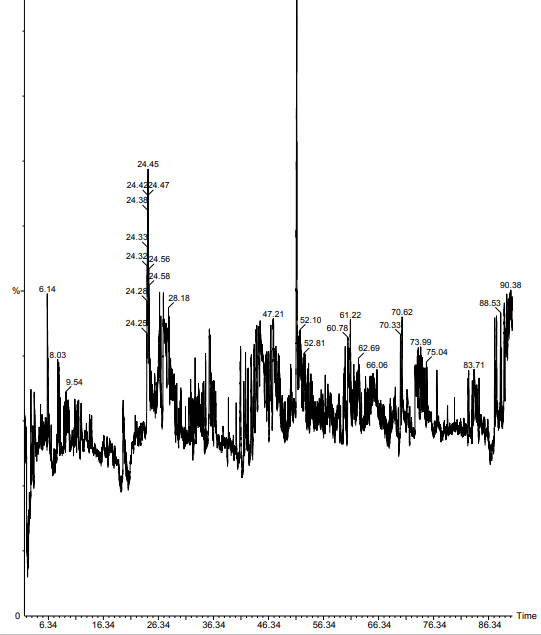


**Abundance (%)**

**Retention time (min)**

S13 Fig

Supplement: S13 Fig — The chromatogram shows major peaks at various retention times that correspond to different compounds. (DOCX) [file pone.0321190.s015.docx]

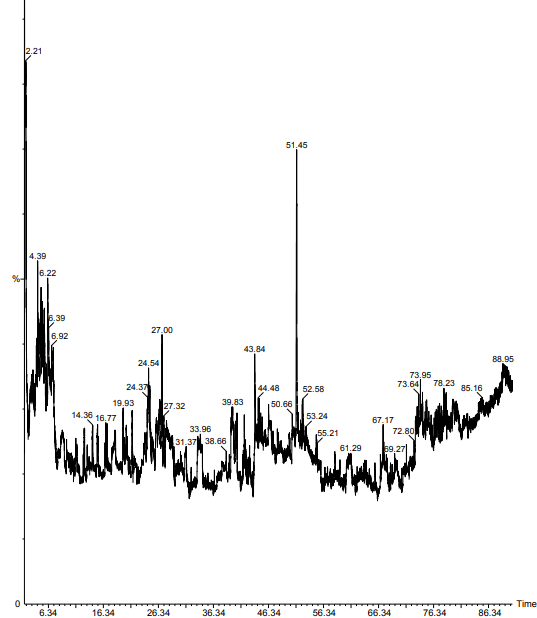


**Abundance (%)**

**Retention time (min)**

S14 Fig

Supplement: S14 Fig — The chromatogram shows major peaks at various retention times that correspond to different compounds. (DOCX) [file pone.0321190.s016.docx]
